# Twenty Years of KSHV

**DOI:** 10.3390/v6114258

**Published:** 2014-11-07

**Authors:** Yuan Chang, Patrick Moore

**Affiliations:** Cancer Virology Program, University of Pittsburgh Cancer Institute, 5117 Centre Avenue, Pittsburgh, PA 15213, USA

**Keywords:** KSHV, Kaposi’s sarcoma, AIDS, virus discovery, history, viral oncogenesis

## Abstract

Twenty years ago, Kaposi’s sarcoma (KS) was the oncologic counterpart to Winston Churchill’s Russia: a riddle, wrapped in a mystery, inside an enigma. First described by Moritz Kaposi in 1872, who reported it to be an aggressive skin tumor, KS became known over the next century as a slow-growing tumor of elderly men—in fact, most KS patients were expected to die with the tumor rather than from it. Nevertheless, the course and manifestations of the disease varied widely in different clinical contexts. The puzzle of KS came to the forefront as a harbinger of the AIDS epidemic. The articles in this issue of Viruses recount progress made in understanding Kaposi’s sarcoma herpesvirus (KSHV) since its initial description in 1994.

Kaposi’s early microscopic studies revealed KS to be a complex tumor composed of disorganized endothelial cell proliferations forming blood-filled vascular clefts but also containing areas of organized micro-neovascularization and often with an inflammatory infiltrate. Pathologists and clinicians became suspicious of whether KS was a “true cancer”: most histopathologic examinations showed no clear demarcation of tumor margins, and the tumor often had a waxing and waning course. Although KS-like spindle cells could be grown in culture from KS lesions, these same cell proliferations could be also generated from blood, and were subsequently shown by Browning to be circulating endothelial precursor cells [1]. Later it would be found that cultured “spindle cells” from KS tumor specimens do not retain the KSHV genome [2].

The epidemiology of KS was complex as well, but proved critical in deciphering the biology of KSHV. Early on, KS was thought to be most common among elderly Ashkenazi Jewish men and later was also reported in isolated European ethnic groups, particularly Sicilians and residents of the Po Valley [3]. By the 1950s, extraordinary rates of KS were recognized to occur in parts of Central and East Africa, where, in some cancer registries, KS was one of the most commonly-reported cancers [4]. Unlike European (or “classic”) KS, KS in Africa (“endemic KS”) could occur in children of both sexes where it had lymphadenopathic dissemination that was nearly always rapidly fatal [5]. Among African adults, KS still showed predisposition to males over females but tended to also be more aggressive than the classic variant. With the development of transplantation treatment regimens and immunosuppression-promoting cancer therapies, a new and striking form of KS emerged in the 1970s [6]. Iatrogenic KS differed dramatically from previous forms since it was highly aggressive, often fatal, and had a more balanced sex ratio. Studies of transplantation registries repeatedly reveal that KS is the most significantly elevated cancer among transplant patients [7]. These odd geographic and epidemiologic patterns suggested to some that KS might be a cancer caused by a virus. 

Emergence of the AIDS epidemic in the early 1980s focused scientific attention on this curious cancer [8]. Previously healthy young men were suddenly being struck by disseminated KS as well as other, previously-rare infections mainly seen among highly immune suppressed transplant patients. The aggressiveness of this AIDS-related cancer left little doubt that it was under immune control. Discovery of the human immunodeficiency virus in 1983–1984 made clear that AIDS is caused by a retroviral ablation of CD4^+^ T cells, but how and why do AIDS patients get this cancer? Epidemiologists described yet again new patterns for “epidemic” or AIDS-KS: the disease occurred almost exclusively among the subset of gay and bisexual men with AIDS (These patterns refer exclusively to North American and European populations. In Africa, there was an explosive epidemic of KS together with the HIV epidemic that affected virtually all populations: children and adults; homosexuals and heterosexuals; and men and women [9]).

By this time, the concept that tumor cells emerge after multiple genetic hits was widely held, and the patterns of KS occurrence remained mysterious. First, KS preferentially occurred among men-who-have-sex-with-men and bisexual male AIDS patients—in fact, so commonly, that the established paradigm requiring multiple genetic hits to generate cancer could not apply. In contrast, KS was relatively rare among equally immune suppressed HIV+ but heterosexual men (such as persons with hemophilia or blood transfusion recipients). Despite the variety of clinical manifestations for KS, all the different forms of KS had indistinguishable pathologic features suggesting it is a single cancer having a common underlying cause.

In a landmark epidemiologic study, Valerie Beral, Harold Jaffe and colleagues sorted out the evolving epidemiology of KS, taking into account not only AIDS-KS patterns but also a century’s worth of observations on KS occurrence prior to the AIDS epidemic [10]. Their conclusion was that AIDS-KS was caused by a sexually transmitted virus that had not yet been discovered since the agent did not fit the clinico-epidemiology patterns for any known virus at that time. The agent could be most efficiently transmitted through homosexual activity (although the precise sex behavior is still unknown) but it would not cause disease unless the infected person also developed severe immune suppression. Further, while HIV is readily transmitted by blood-borne infection, the putative KS agent is not. Hence, transfusion recipients and persons with hemophilia could develop AIDS from HIV infection but would be at a low risk for AIDS-KS. Similar to some other viruses (e.g., hepatitis B virus), the agent might be transmitted through nonsexual mechanisms (that also still remain poorly understood) and developing country might have entirely different transmission patterns from those of developed countries. Geographically, the KS agent should be hyperendemic in sub-Saharan Africa, less so in nearby Mediterranean countries and lowest in North America, Northern Europe and Eastern Asia. Beral and Jaffe’s study inferred that the KS agent should be uncommon in the general U.S. population.

By 1994, years of research had failed to reveal the identity of this agent. Over 20 different viruses, bacteria and environmental exposures had been proposed, but none of them fit the established patterns for KS. Many of these agents could be easily dismissed—the idea that recreational nitrite poppers act as mutagens for KS seemed potentially plausible for a subset of club-culture AIDS patients but certainly did not help explain KS among more sedentary gay men infected with HIV nor Africans or transplant patients. Other agents, such as cytomegalovirus (CMV), required years of replicative studies before the consensus conclusion was reached that CMV is not the cause for KS. HIV itself was promoted to cause KS by inducing inflammation. This inadequately explained KS occurring prior the AIDS epidemic or lack of KS among blood-recipients with AIDS and high HIV loads. The early 1990s was also a period of turmoil and frustration for scientists working on HIV/AIDS. Protest marches against AIDS and sit-in demonstrations at the U.S. Food and Drug Administration were commonplace. Enormous amounts had been learned about HIV, but death rates from AIDS continued to rise and the origins for KS remained obscure.

## Discovery of KSHV

In early 1993, we had just moved to New York City, and the Department of Pathology at Columbia University generously provided a 100 square foot laboratory and $20,000 start-up funding to Chang to perform clinical neuropathology research. Moore had been an epidemiologist at the Centers for Disease Control working on meningitis epidemics and refugee disasters in Africa. He took a job in the New York City Department of Health. We had been married for five years but had never worked together.

At this time, several events contributed to our interest in searching for a KS pathogen. Genome scanning techniques then held promise for identifying large chromosome regions mutated in tumors, and Chang became interested in using these techniques. Moore had been involved in the control of a 1991 hemorrhagic fever epidemic in Nigeria. The cause turned out to be a variant yellow fever virus that was undetectable on standard diagnostic tests but could have been prevented with a cheap and effective vaccine had it been identified early in the outbreak.

Molecular biology techniques developed for non-directed genomic searching—such as representational difference analysis (RDA) [11] described by the Lisitsyns and Wigler—seemed promising for identifying foreign DNA from agents in outbreak settings as well. RDA is a PCR-based method to kinetically-enrich for novel genomic differences between two complex tissue samples. At the time, most new viruses were identified by direct animal or cell culture inoculations and serologic tests but these traditional techniques had clearly been unsuccessful in finding a KS agent. Lisitsyn *et al.* generously provided a detailed RDA protocol but our first pilot project to RDA isolate lambda phage DNA spiked into a human tissue sample failed. The costs for this experiment in buying Taq polymerase alone nearly broke our meagre start-up budget. We did not have other options than to try again using an actual KS tumor without the typical optimization that normally would have been done.

**Figure 1:**
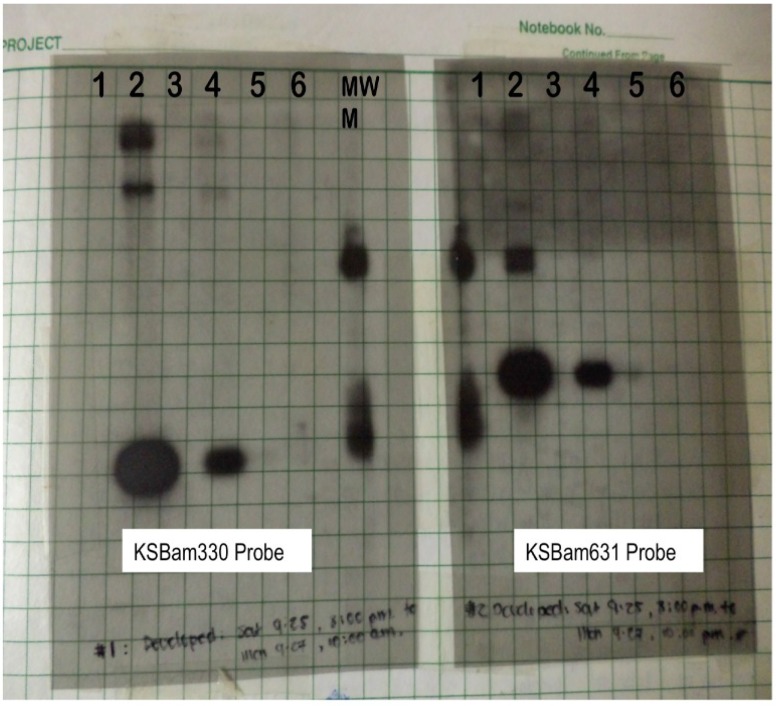
Southern blotting with probes using cloned RDA products. Left panel probed with the KSBam330 band and right panel probed with the KSBam631 band. Lanes 1, 2, 3 were DNA extracted from AIDS lymphomas; #2 is a PEL that strongly hybridized with both probes. A cell line established previously from this lymphoma was subsequently named BC-1. Lane #4 was DNA extracted from the KS lesion used for RDA tester and Lane #5 contained DNA the same patient’s healthy control skin that was for the RDA control driver. Lane #6 was PBMC DNA from another patient with KS lesions.

In early May of 1993, Anna Batistatou, then a resident in the Department of Pathology at Columbia, alerted us to her autopsy on a middle-aged man with AIDS-KS. This case turned out to be unusual for several reasons: firstly, the concern of contracting HIV infection from a slipped scalpel during an AIDS autopsy had driven this procedure to a trickle by 1993. Batistatous’s AIDS autopsy case would be one of the last ones performed at Columbia for several years and, although we did not know it at the time, our major local source for KS tissues was soon to be gone. Secondly, we later found that this patient’s KS lesions had the highest KSHV load of any KS tumors that we subsequently collected. A KS lesion and a control skin sample were taken for RDA analysis, which was performed by Chang and Melissa Pessin, a rotating Pathology resident. The iterative protocol began on August 12 and four RDA bands were isolated on September 7 and cloned by September 15, 1993.

Given the possibility for PCR contamination, we chose to analyze the bands by the more time consuming, but dependable, Southern blotting technique. Detection would not be a problem since the KS agent should be present in every tumor cell. Further, a less sensitive technique would have the advantage of reducing false-positive results from a coincidental but non-causal infection. The films were developed on September 27, providing the first hint that something new might be present (Figure 1). The patient’s KS tumor was positive for two of the RDA fragments (the other two fragments appeared to be human DNA) and his control tissue was negative. We couldn’t ask for a more satisfying result in the patients samples—the DNA fragments were not a human polymorphic sequence artificially amplified by the RDA process and both probes were positive in the same pattern. Further, whatever agent was present in the sample was abundant and made of DNA. 

But there were mitigating concerns. We had earlier asked another Pathology resident, Anne Matsushima, for non-KS tissue samples from AIDS patients to use as negative controls. This would help ensure that we hadn’t isolated some opportunistic but unrelated pathogen DNA abundant in AIDS patients. She provided three AIDS lymphoma samples; one (Figure 1, Lane 2) had a signal that was an order of magnitude more intense than the AIDS KS tissue. The chance that Beral-Jaffe’s agent actually causes KS and is also present in 33% of randomly selected AIDS-lymphomas—at much higher copy number than KS—seemed extremely unlikely.

With this paltry information and two unknown DNA sequences in hand, we began a collaboration with Ethel Cesarman and Dan Knowles. Out of 27 KS samples from the Hematopathology tissue bank, 25 were positive for the RDA fragments on blinded testing together with other control non-KS tissues from AIDS and non-AIDS patients. After breaking the code, we double-checked the two negative samples and found one was comprised of degraded DNA and the other was not actually KS but had been mislabeled. Slowly, the pattern began to emerge that actually did fit the Beral-Jaffe agent. The possibility that we were misinterpreting due to some trivial but subtle mistake was a constant concern. So there was palpable relief when we learned from Robin Weiss and Thomas Schulz in the UK, who agreed to test their own KS samples, that the same results were independently reproducible [12]. As it turned out, the one positive AIDS lymphoma DNA we obtained from Anne Matsushima happened to be a rare specimen from a body cavity-based lymphoma (subsequently renamed to primary effusion lymphoma, PEL), now known to harbor 40–80 times more KSHV DNA than KS tumors. Of 193 AIDS lymphoma samples Cesarman later examined, only 8 were positive for KSHV DNA and all of these were PEL [13]. The only control tissues that were also occasionally positive were hyperplastic lymph nodes from AIDS patients. In August 1995, Soulier and colleagues would report that AIDS-related Castleman’s tissues—often presenting as lymph node hyperplasias—are also commonly infected by KSHV [14].

**Figure 2:**
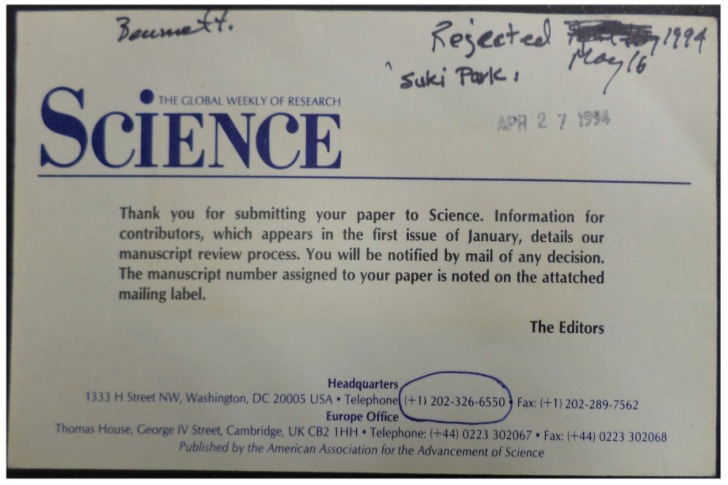
The first KSHV description was submitted to the journal Science on April 27, 1994 and summarily rejected. More sequencing and conversations with the editor were required for the paper to be reconsidered.

Another four more months passed before the RDA fragments could be shown to be from a new human herpesvirus, similar to but distinct from any known herpesvirus. The next 15 months were filled with nearly nonstop work screening lambda phage libraries, sequencing the virus, isolating it in PEL cell culture, developing serologic tests and testing more tumor specimens. All successful tumor viruses go through a peer-review version of the Kübler-Ross stages: first there is shock and denial, then anger, next bargaining and depression, and, finally, acceptance (Figure 2). The initial KSHV description was finally published in December 1994 [15], which began another stage of exciting and sometimes stormy [16] research on this peculiar virus. Over the past 20 years, the combined efforts of an extraordinary and talented group of scientists around the world has forced KS to begin to give up answers to some of its enigmatic and mysterious riddles.
